# Clinical Validation of a Video-Otoscopy-Based Medical Device for the Remote Diagnosis of Ear Complaints

**DOI:** 10.3390/s25030758

**Published:** 2025-01-27

**Authors:** Ádám Pannonhalmi, Bálint Posta, Ádám Perényi, László Rovó, Balázs Bende, Gábor Katona, Ildikó Csóka, Lajos Kemény, László Szakács

**Affiliations:** 1 Department of Dermatology and Allergology, University of Szeged, Korányi Alley 6, H-6720 Szeged, Hungary; pannonhadam@gmail.com (Á.P.); bende.balazs@med.u-szeged.hu (B.B.); kemeny.lajos@med.u-szeged.hu (L.K.); 2 Department of Oto-Rhino-Laryngology and Head-Neck Surgery, University of Szeged, Tisza Lajos Blvd. 111, H-6725 Szeged, Hungary; posta.balint@med.u-szeged.hu (B.P.); perenyi.adam@med.u-szeged.hu (Á.P.); office.orl@med.u-szeged.hu (L.R.); 3 Institute of Pharmaceutical Technology and Regulatory Affairs, Faculty of Pharmacy, University of Szeged, Eötvös Street 6, H-6720 Szeged, Hungary; katona.gabor@szte.hu (G.K.); csoka.ildiko@szte.hu (I.C.)

**Keywords:** telemedicine software, eHealth, video-otoscopy, medical device, remote diagnostics

## Abstract

Telemedicine brings several benefits to patients, healthcare providers, and the wider society, including reductions in the need for hospitalizations or readmissions, as well as in overall healthcare costs and the length of inpatient stay. In addition, these services may provide psychological benefits to patients, including excellent satisfaction and medication adherence. The present study aimed to investigate an in-house-developed otorhinolaryngologic remote diagnostic system (mobile app). The basis of the comparison was the incidence between the diagnoses and therapies made by remote diagnosticians and on-site specialists based on static images and videos captured by a smartphone otoscope device. In the study, 103 patients were involved. After registering demographic data, the telemedicine software was evaluated by comparing the matching of physically established diagnoses and/or therapies with remotely established diagnoses and/or therapies. The most remarkable result was in concordance with the diagnoses, with 79 matches identified of the 103 cases examined; the rate of the matching cases was 76.7% (95% CI: 68.5–84.9%). These results support that telemedicine-based otorhinolaryngological remote diagnostics could play a significant role in future healthcare.

## 1. Introduction

According to the World Health Organization (WHO) demographic data, in most parts of the world, the population has been rapidly aging in the past 20 years, which has placed additional burdens on the provision of healthcare services. The ageing population forms a captive market for the medical devices industry due to the rising demand for home care solutions for the elderly. Advancements in medical device technology have significantly increased the number of conditions that can be managed in a home care setting. To capitalize on this strong demand and growth, many manufacturers have emerged with a rapid market entry goal to produce consumer medical devices [1].

The growing medical device market required a properly regulated quality assurance system; therefore, the Medical Devices Directive (MDD), as the first regulatory requirement, was established and applied to all medical devices sold on the European market [2]. The MDD is an important legislation for medical device manufacturers because it provides a framework to ensure their product complies with European standards [3]. However, MDDs lacked certain aspects, and there was also an increased demand from consumers for transparency of medical equipment and device technical information; it was necessary to create a regulation system, i.e., the Medical Device Regulation (MDR) [4]. One of the key distinctions between MDR and MDD is that MDDs do not include additional requirements beyond what items are already covered by the EU legislation (such as a CE marking). Another major drawback of MDD was that telemedicine solutions, such as mobile apps that patients use to monitor their health, did not exist at its creation, and Software as a Medical Service (SaMD) was not even a concept then [5].

Telemedicine brings several benefits to patients, healthcare providers, and the wider society, including reductions in the need for hospitalizations or readmissions, as well as in overall healthcare costs and the length of inpatient stay. In addition, these services may provide psychological benefits to patients, including greater rates of satisfaction and medication adherence [6]. Moreover, several healthcare areas have successfully adopted telemedicine during the COVID-19 pandemic [7]. eHealth, telehealth, and telemedicine solutions can be used interchangeably, providing convenient, low-cost, and accessible health-related information and communication remotely using internet-based technologies [8]. SaMD is a software that, alone or in combination, serves the purposes specified in the MDR or the definition of “medical device” in the In Vitro Diagnostic Medical Devices Regulation [9]. The MDR classification reflects the risk of harm that a medical device poses. Standalone Software will be classified based on intended purpose and risk assessment, not purpose alone. Using these systems can benefit both the patient and the care institution; in this structure, the patient does not need to travel to a distant specialist care institution, and the care institution can operate more efficiently.

**Figure 1:**
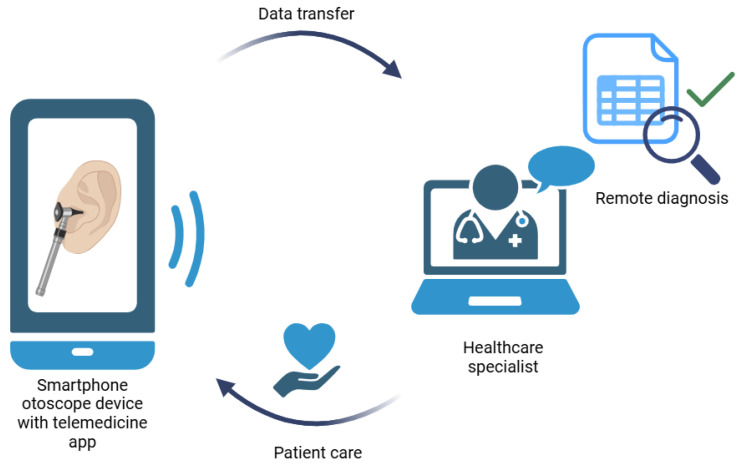
Schematic presentation of otorhinolaryngological remote diagnosis and management.

Telemedicine applications have been successfully used by various segments of the clinic for years. The most common areas of application include teledermatoscopy, teleradiology, telemental health and teleopthalmoscopy. Increasingly, healthcare units are utilizing teleradiology, telecardiology and tele-intensive-care-unit services that monitor patients 24 h per day and alert on-site healthcare providers [10]. Telemedicine strategies have been shown to play an increasing role in ear disease diagnosis. The use of telemedicine solutions is beneficial for both the patient and the otorhinolaryngologist: as the medical examination can be performed even at home or in primary care, the waiting time and the burden on the hospital care system can be reduced, and the diagnosis can be made faster, even in case of pandemic situations [11]. With the advance of technology in the field of otorhinolaryngology, several diagnostic devices have evolved over time. The development of the video otoscope brought the possibility of using the smartphone as an important diagnostic tool in clinical practice. Smartphone otoscopes can record images of comparable diagnostic utility to conventional otoscopy coupled with a custom software app, enabling the capture of video and static images in real time that may be stored and transmitted to healthcare specialists using wireless communication for remote diagnosis and management (Figure 1) [12]. Using them, healthcare specialists can quickly and easily screen out serious symptoms such as bleeding and pus, which need emergency intervention. Previously, several studies already discussed the clinical assessment of video-otoscopy in remote diagnosis by specialists [13,14,15,16,17,18,19,20]. The novelty of our work lies in focusing the utilization of telemedicine at the primary care level. The burden of higher (III) progressiveness levels can be reduced in this way. The smartphone and the otoscope that can be connected to it are a relatively inexpensive, easily accessible otorhinolaryngology diagnostic tool in the hands of the primary care provider, which greatly supports diagnostics and documentation, and last but not least, helps communication between the primary care provider and the specialist. By remote education of family doctors, their gatekeeper role could be increased. Moreover, it can be a suitable tool for family doctors to examine bedridden patients at home.

The purpose of this open prospective study was to test an in-house-developed otorhinolaryngologic remote diagnostic system (mobile app) by comparing the agreement between diagnoses and therapies made by remote diagnosticians and on-site specialists based on static images and videos captured by a smartphone otoscope device, in order to investigate the possibility of implementing telemedicine between primary care providers and specialists.

## 2. Materials and Methods

### 2.1. Participants of the Study

The study was conducted in accordance with the Declaration of Helsinki. It was approved by the Medical Research Council and the National Scientific and Ethical Committee (OGYÉI/1422/2020). Written informed consent was obtained from the patients or their parents/guardians before the study and participation was voluntary. The study included 103 patients aged 0–100 years with otorhinolaryngological complaints, and who have consented to the examinations. From January to May 2020, data were collected from patients with otorhinolaryngological complaints admitted at the Department of Oto-Rhino-Laryngology and Head-Neck Surgery, Faculty of Medicine, University of Szeged, based on the inclusion and exclusion criteria. The patients with ear complaints (mainly but not limited to earache, discharge from the ear, itching, and cerumen accumulation) were involved in the study, who communicated well with the investigator and could understand and comply with the requirements of the protocol, and signed the informed consent. The exclusion criterion of the study was, on the one hand, the patient’s withdrawal of informed consent at any time after being informed and signing, thereby suspending his or her participation orally or in written form. On the other hand, patients with poor general conditions, traumatic events or symptoms that may be directly life-threatening were excluded from the study, as determined by the investigator (e.g., cerebrospinal fluid leakage or massive bleeding). Further exclusion reason was any medical condition which, in the investigator’s opinion, may have compromised the patient’s health and/or contraindicate the conducting of the study, or the patient’s participation was suspended for any reason according to the investigator.

### 2.2. Applied Telemedicine Device and Software

**Figure 2:**
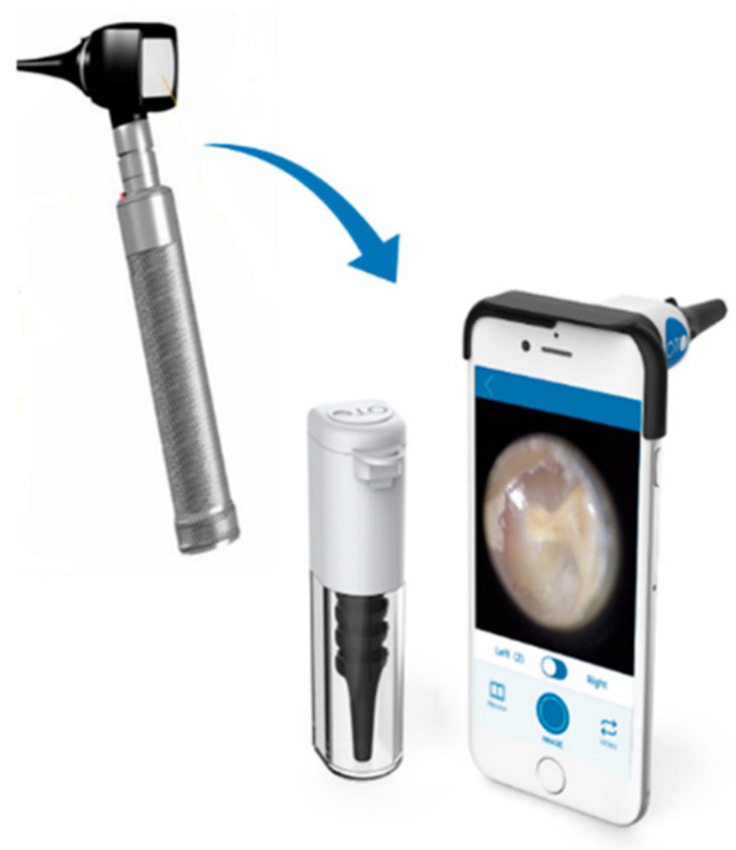
Smartphone otoscope device [21].

The telemedicine software developed by the University of Szeged was used for the study, which supports the transmission and analysis of otoscopic images and clinical data. The device consisted of a CE-marked optical unit applied (Cupris TYM smartphone otoscope device, Cupris Ltd., Somerset, UK) to a CE-marked smartphone and the software that operates the system, and was the subject of the test, as well as a detailed user guide. The device was used to record videos of the external auditory canal and the tympanum, and to record clinical data (symptoms, complaints) (Figure 2). The phone application transmitted the data to the examining physician, who analyzed the data, made a diagnosis and therapeutic recommendation and arranged the following visit.

### 2.3. Study Protocol

**Figure 3:**
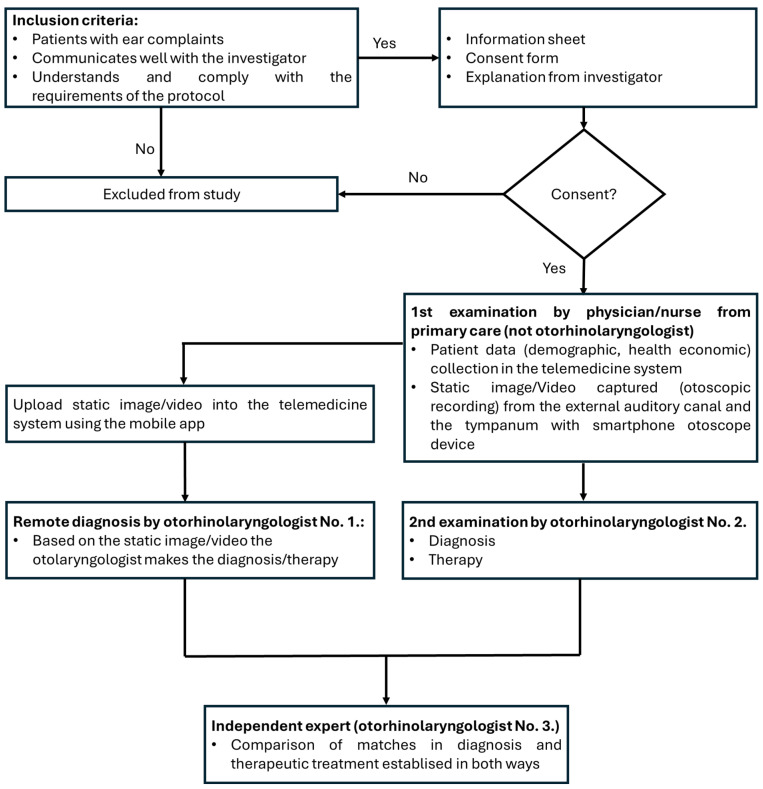
Study protocol flow diagram.

Patients who visited the Department of Oto-Rhino-Laryngology and Head-Neck Surgery or outpatient clinic were informed about the nature of the study before signing the informed consent forms. Then, the data necessary for the demographic data of recruited patients were recorded in the telemedicine software by a physician/nurse from primary care (who is not an otorhinolaryngologist specialist). After that, a static image or video (otoscopic recording) of the external auditory canal and the tympanum was recorded, uploaded into the telemedicine system and shared with a specialist (otorhinolaryngologist no. 1.) for selecting the proposed diagnosis in the telemedicine system based on the telemedicinal data. Parallel to that, the patient was physically examined by another independent specialist (otorhinolaryngologist no. 2.) who recorded the anamnesis with the same questions included in the telemedicine system, defined diagnosis and recommended therapy from the optional list that was included in the telemedicine system. Finally, an individual expert (otorhinolaryngologist no. 3.) compared whether the diagnoses and the treatments made by the two ways were matching. In the case of the match, only one was assessed; however, if there was a difference, both were assessed. The study design algorithm is shown in Figure 3.

### 2.4. Data Analysis

The main aim of the study was to compare the consistency of the diagnoses and therapeutic treatments made by remote diagnostics and on-site specialists. The primary target variable was the “ratio of matches”, which defines the percentage result of matches compared to total number of cases. Principally, the diagnosis was selected based on the International Statistical Classification of Diseases and Related Health Problems (ICD). The two study results are considered matching if the same diagnosis was selected from the drop-down menu and the main group (first level) of therapy was the same (not at the active pharmaceutical ingredient and dose level), and was confirmed by the independent expert.

For demographic data evaluation, descriptive statistics provided the number of cases, the mean value, the standard deviation, the minimum, the median and the maximum values. In the category variables, the number of cases and the frequency of occurrence were given.

The primary efficacy analysis examined the ratio of the matching therapies and diagnoses. The ratios and the 95% confidence intervals are given, and the Wald test was used to study the matches above 90%. The statistical significance level was 0.05. The secondary efficacy analysis examined the ratio of matching therapies and matching diagnoses. The ratios and the 95% confidence intervals are given, and the Wald test was used to study the matches above 90%. The statistical significance level was 0.05. Descriptive statistical methods were applied for further analyses (same diagnosis, different therapy, matches per each optional diagnosis).

The ratio of different therapies and diagnoses and the ratio of matching and overlapping therapies and diagnoses were studied. These ratios and the 95% confidence intervals are provided.

## 3. Results

### 3.1. Demographic and Basic Characteristics

One hundred and three (103) patients were enrolled in the study: 54 females (52%) and 49 males (48%) with an average age of 45.5 ± 19.57 years (range, 15–96 years) with a median age of 43.5 years. The cohort was predominantly composed of adults, the median age was 43.5 years. Regarding the travel distance of the patients, we found that the involved patients traveled an average of 12.9 km between their residence and the clinic, most of them by car (31.1%). Moreover, due to the otorhinolaryngological examination, 39.8% of the patients had lost working hours, an average of 2.7 h (SD, 1.85). Telemedicine solutions offer a suitable alternative to reduce travel distance, time and costs, as well as the loss of working hours. Using a smartphone otoscope device, the patient or their relative was able to capture static images or video from the affected ear canal at home easily and comfortably, and it provided for establishing a remote diagnosis to the healthcare specialist through the telemedicine software.

### 3.2. Primary Analysis

#### Number of Matches in the Diagnoses and Therapies Together

The matching of the diagnoses and therapies was investigated simultaneously during the primary analysis of the telemedicine software. However, full matches were only found in 56 of the 103 cases examined, and the rate of the matching cases was 54.4% (95% CI: 44.7–64.0%). The ratio of the matching cases was significantly lower than the expected 90% matching (Wald test, *p* < 0.001) (Table 1). Therefore, in the secondary analysis, the matching of the diagnoses and therapies was investigated separately to determine which case resulted in a more remarkable difference.

### 3.3. Secondary Analysis

#### 3.3.1. Number of Matches in the Diagnoses Individually

During the secondary analysis of telemedicine software, the matching in diagnoses was first investigated. The results showed that matching diagnoses were found in 79 of the 103 investigated cases, and the rate of the matching cases was 76.7% (95% CI: 68.5–84.9%). The ratio of the matching cases was still significantly lower than the expected 90% matching (Wald test, *p* < 0.001) (Table 2). However, these results indicated that the telemedicine software is suitable for remote diagnostics. A higher number of cases may increase the rate of matching cases.

#### 3.3.2. Number of Matches in the Therapies Individually

In the other part of the secondary analysis, for the matching therapies, it was revealed that matching therapies were found in 61 of the 103 investigated cases; the rate of the matching cases was 59.2% (95% CI: 49.7–68.7%). The ratio of the matching cases was significantly lower than the expected 90% matching (Wald test, *p* < 0.001) (Table 3). This result indicates that the number of matchings in primary analysis was lower because of the relatively high number of failed matches in therapies.

#### 3.3.3. Number of Matches in the Diagnoses in the Case of Different Therapies

In further analysis, the data of those 79 volunteers were taken into consideration, in which case matches in diagnoses were found (Table 4). In the case of 56 patients (70.9%), there was also a match in therapy, while only 23 cases (29.1%) were found in different therapies. Table 4 shows the summary of matching and non-matching diagnoses and therapies.

#### 3.3.4. Matching by Optional Diagnosis

Matching in diagnoses and therapies was also investigated in the case of the optional diagnosis provided by telemedicine software. The ratio and the 95% CI of the matching diagnoses and the matching therapies for each optional diagnosis are shown in Table 5. It was revealed that in the case of optional diagnoses with the highest case number (5–30), such as Eustachian salpingitis, Impacted cerumen, Otitis externa maligna, and Otitis externa, the rate of matching in diagnoses was higher than 75%, which confirms the validation of the software for remote diagnostics; however, in the case of optional diagnosis with a low case number (1–4), there was high deviation. A similar tendentious conclusion cannot be drawn when investigating the matching of therapies.

### 3.4. Additional Analysis

#### Overlapping in Diagnoses and Therapies

**Figure 4:**
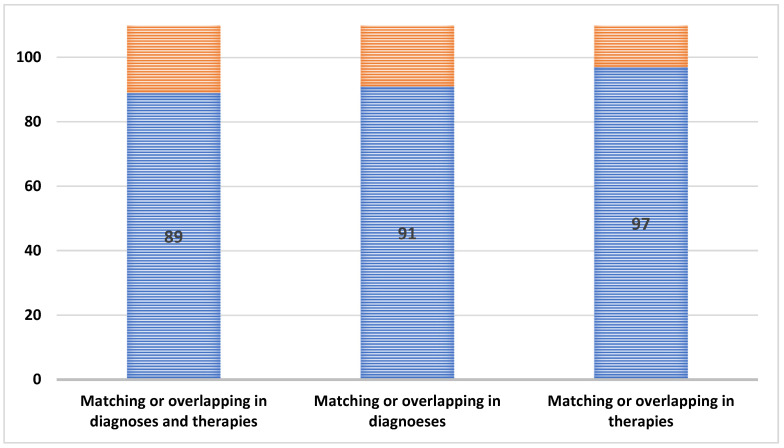
Number of cases with matching or overlapping diagnoses and/or therapies (n = 103).

When the diagnosis and therapy did not match, overlapping between the diagnoses and the therapies was investigated (Figure 4). The diagnoses and therapies showed a separate matching or overlapping in 89 cases from the investigated 103 ones. The matching or overlapping diagnoses and therapies ratio was 86.4% (95% CI: 79.8–93.0%). In the case of non-matching diagnoses, matching or overlapping was found in 91 of the 103 cases examined. The matching or overlapping diagnoses ratio was 88.3% (95% CI: 82.2–94.5%). In the case of non-matching therapies, matching or overlapping was detected in 97 cases of the investigated 103. The matching or overlapping therapy ratio was 94.1% (95% CI: 89.7–98.7%).

## 4. Discussion

Telemedicine systems can be beneficial for both the patient and the healthcare institution. The most significant advantage for patients may be that they do not need to travel to a distant specialist care unit, while the healthcare institution can operate more efficiently. The present study aimed to compare the agreement between the diagnoses and therapeutic treatments made by remote diagnosticians and on-site specialists. In the study, 103 patients were involved; all of them completed the study according to the protocol. Thus, the total number of population elements was 103 in each analysis.

The average age of the patients was 45.5 ± 19.57 years; the youngest patient was 15, and the oldest was 96 years old. Patients included in the study traveled an average of 12.9 km to their specialist care, most of them by car (31.1%). Moreover, 41 participants were absent from work, with 2.7 working hours lost on average.

The primary target variable was the investigation of the ratio of matches in diagnoses and therapies. Two study results were considered to match if the diagnosis and the main treatment group were the same. The most remarkable result was obtained in the case of the matching diagnoses, where 79 matches were found of the 103 investigated cases; the ratio of the matching cases was less than 90% based on the Wald test. According to medical opinion, this may be because the device is only suitable for visual diagnostics, which does not give a complete opinion of the ear canal due to the high frequency of wall cerumen (earwax) presence. Although the image and video documentation provided by the device was of acceptable quality, in many cases, it was not suitable for assessing subtle abnormalities. While this circumstance does not make a real difference in the patient’s treatment, it does result in different diagnoses. This can be significantly reduced with the development of otoscope and phone hardware, which improves the resolution or image quality and can lead to a much clearer and more certain diagnosis. Another possibility for development is the integration of artificial intelligence-based algorithms to increase the accuracy of the analysis and reduce the number of incorrect diagnoses. Nowadays, with the update of the system and the use of new mobile devices and higher resolution otoscopes, better image quality (4k or better) can be achieved. The project required software development and validation by a statistics company, which entailed costs, and from an administrative point of view, clinical trial management, clinical trial staff (doctors, nurses) and internal monitoring were required. However, the material costs (software development and statistical testing costs) can be reduced over time, after validation and a successful pilot study, so it can be easily implemented into the patient care system. Remote otoscopy would primarily help primary care, since a wide range of expensive otorhinolaryngologic diagnostic tools is not available to general practitioners. However, with the help of a simple smartphone, application and a connectable otoscope, appropriate diagnostics and documentation can be achieved, which can increase the gatekeeper role of general practitioners.

No adverse event was reported during the study, so no safety assessment was carried out. All the involved patients completed the study in accordance with the study protocol, and no deviation from the study protocol was recorded.

The physicians participating in the study found that most patients accepted the participation well and were happy to participate. They were interested in the study and welcomed the new opportunity. They even accepted the need for administration because they thought their involvement was forward-looking. The data collected and the experience gained during the study contributed to the application of telemedicine methods in patient care.

## 5. Conclusions

The present study reported on the clinical validation of an in-house-developed otorhinolaryngologic remote diagnostic system (mobile app). The validation was based on comparing the incidence of diagnoses and therapies made by remote diagnosticians and on-site specialists via the mobile application, which used static images and videos recorded with a smartphone otoscope device. Using a smartphone otoscope device, the patient or their relative was able to capture static images or video from the affected ear canal at home easily and comfortably, and provide remote diagnostics to the healthcare specialist through telemedicine software. The experimental data also supported that establishing a remote diagnosis could significantly affect future healthcare. The most remarkable result was in concordance with the diagnoses, with 79 matches identified of the 103 cases examined; the rate of the matching cases was 76.7% (95% CI: 68.5–84.9%). This result can be improved with the development of mobile devices and higher-resolution otoscopes, achieving better image quality (4K or better), which can lead to a much clearer, more certain diagnosis. Another possibility for overcoming the limitations is the integration of artificial intelligence-based algorithms to increase the accuracy of the analysis and reduce the number of incorrect diagnoses. Last, but not least, it would be worthwhile to expand the study to more subjects to obtain relevant statistical results. The implementation of remote otoscopy would primarily help primary care, since a wide range of expensive otorhinolaryngologic diagnostic tools is not available to general practitioners. However, with the help of a simple smartphone, application and a connectable otoscope, appropriate diagnostics and documentation can be achieved, which can increase the gatekeeper role of general practitioners. Our future plans are to further develop the telemedicine software by adding a long-term follow-up module that can also analyze comorbidities, as well as other modules adapted to the telemedicine system that help with communication between the general practitioner and the specialist, data management and effective remote diagnosis.

## Figures and Tables

**Table 1:** Matching diagnoses and therapies—test statistics.

One-Sample Proportion Test									
	Test Type	Observed				Asymptotic Standard Error	Z	Significance	
		Success	Trials	Proportion	Test Value			One-Sided *p*	Two-Sided *p*
Are the diagnoses and the therapies matching? = Matching	Wald	56	103	0.544	−0.356	0.049	−7.260	<0.001	<0.001
	Wald (Continuity Corrected)	56	103	0.544	−0.356	0.049	−7.161	<0.001	<0.001

Test Value = 0.9.

**Table 2:** Matching diagnoses—test statistics.

One-Sample Proportion Test									
	Test Type	Observed				Asymptotic Standard Error	Z	Significance	
		Successes	Trials	Proportion	Test Value			One-Sided *p*	Two-Sided *p*
Are the diagnoses matching? = Matching	Wald	79	103	0.767	−0.133	0.042	−3.193	<0.001	0.001
	Wald (Continuity Corrected)	79	103	0.767	−0.133	0.042	−3.077	0.001	0.002

Test Value = 0.9.

**Table 3:** Matching therapy—test statistics.

One-Sample Proportion Test									
	Test Type	Observed				Asymptotic Standard Error	Z	Significance	
		Successes	Trials	Proportion	Test Value			One-Sided *p*	Two-Sided *p*
Are the therapies matching? = Matching	Wald	61	103	0.592	−0.308	0.048	−6.356	<0.001	<0.001
	Wald (Continuity Corrected)	61	103	0.592	−0.308	0.048	−6.256	<0.001	<0.001

Test Value = 0.9.

**Table 4:** Matching diagnoses and therapies—cross table.

			Are the Therapies Matching?		Total
			Non-Matching	Matching	
Are the diagnoses matching?	Non-matching	Count	19	5	24
		% (Are the diagnoses matching?)	79.2%	20.8%	100.0%
		% (Are the therapies matching?)	45.2%	8.2%	23.3%
	Matching	Count	23	56	79
		% (Are the diagnoses matching?	29.1%	70.9%	100.0%
		% (Are the therapies matching?)	54.8%	91.8%	76.7%
Total		Count	42	61	103
		% (Are the diagnoses matching?)	40.8%	59.2%	100.0%
		% (Are the therapies matching?)	100.0%	100.0%	100.0%

**Table 5:** Diagnosis and therapy matching ratio by optional diagnoses.

Optional Diagnosis	Are the Diagnoses Matching?				Are the Therapies Matching?			
	Matching				Matching			
	N	Valid N%	95.0% CI Lower	95.0% CI Upper	N	Valid N%	95.0% CI Lower	95.0% CI Upper
Perforation of the tympanic membrane, unspecified	2	100.0%			2	100.0%		
Other otitis externa	1	100.0%			1	100.0%		
Other	2	40.0%	9.4%	79.1%	1	20.0%	2.3%	62.9%
Otalgia	1	33.3%	3.9%	82.3%	1	33.3%	3.9%	82.3%
Eustachian salpingitis	12	75.0%	50.9%	90.9%	7	43.8%	22.2%	67.4%
Tinnitus	3	75.0%	28.4%	97.2%	3	75.0%	28.4%	97.2%
Hearing loss, unspecified	3	50.0%	16.7%	83.3%	5	83.3%	44.2%	98.1%
Acute tympanic membrane inflammation	1	100.0%			0	0.0%		
Acute suppurative otitis media	1	25.0%	2.8%	71.6%	1	25.0%	2.8%	71.6%
Acute serous otitis media	4	36.4%	13.7%	65.2%	3	27.3%	8.3%	56.5%
Foreign body in the ear	3	100.0%			3	100.0%		
Sensorineural hearing loss—unspecified	0	0.0%			1	100.0%		
Chronic serous otitis media	0	0.0%			1	33.3%	3.9%	82.3%
Chronic tubotympanic suppurative otitis media	1	33.3%	3.9%	82.3%	1	33.3%	3.9%	82.3%
Impacted cerumen	26	83.9%	68.2%	93.6%	27	87.1%	72.2%	95.5%
Otitis media, unspecified	1	10.0%	1.1%	38.1%	4	40.0%	15.3%	69.6%
Disorder of external ear, unspecified	1	50.0%	6.1%	93.9%	1	50.0%	6.1%	93.9%
Nonsuppurative otitis media, unspecified	0	0.0%			1	100.0%		
Otitis externa maligna	5	83.3%	44.2%	98.1%	3	50.0%	16.7%	83.3%
Otitis externa, unspecified	30	78.9%	64.2%	89.5%	16	42.1%	27.5%	57.9%
Noise effects on the inner ear	0	0.0%			1	100.0%		

## Data Availability

Data are available on request to the corresponding author.
